# Host–anellovirus interactions in an island ecosystem: non-human primates and rodents in Madagascar harbour diverse, rich anellovirus populations

**DOI:** 10.1099/mgen.0.001681

**Published:** 2026-04-09

**Authors:** Elise N. Paietta, Rachel A. Johnston, Santatriniaina F. Randrianarisoa, Camille M.M. DeSisto, Simona Kraberger, Darren Martin, Tahina T. Razanamahenina, Antsa Ramboninarimalala, Jean-Baptiste Velontsara, Toussaint G. Raherinirina, Victor Rasendrinirina, Laurent Raveloson, Yanting Luo, Nina L. Finley, Eric Baitchman, Anne D. Yoder, Arvind Varsani

**Affiliations:** 1 The Biodesign Center for Fundamental and Applied Microbiomics, Center for Evolution and Medicine, School of Life Sciences, Arizona State University, Tempe, AZ 85287, USA; 2 Department of Biology, Duke University, Durham, NC 27708, USA; 3 Zoo New England, Boston, MA 02121, USA; 4 Mahaliana Labs SARL, Amboditsiry, Antananarivo 101, Madagascar; 5 Rice Sustainability Institute, Rice University, Houston, TX 77005, USA; 6 Computational Biology Division, Department of Integrative Biomedical Sciences, Institute of Infectious Diseases and Molecular Medicine, University of Cape Town, Observatory, Western Cape, South Africa; 7 Department of Zoology and Animal Biology, University of Antananarivo, Antananarivo, Madagascar; 8 Centre ValBio, Ranomafana, Madagascar; 9 Health In Harmony-Fahasalamana Mirindra, Farafangana, Madagascar; 10 Department of Molecular Genetics and Microbiology, Duke University, Durham, NC 27710, USA; 11 Department of Cell Biology, Duke University, Durham, NC 27710, USA; 12 University Program in Genetics and Genomics, Duke University, Durham, NC 27710, USA; 13 Department of Disease Control, London School of Hygiene and Tropical Medicine, London, WC1E 7HT, UK; 14 Structural Biology Research Unit, Department of Integrative Biomedical Sciences, University of Cape Town, Cape Town 7925, South Africa

**Keywords:** *Anelloviridae*, black rat, house shrew, mouse lemur, Webb’s tufted-tailed rat, woolly lemur

## Abstract

Anelloviruses are circular, negative-sense single-stranded DNA viruses that have remarkable diversity and ubiquity across mammals. However, few studies have attempted to determine anellovirus diversity and dynamics across a biodiverse landscape. Madagascar offers a unique opportunity to study anellovirus diversity, with speciose, endemic mammalian lineages that have evolved in geographic isolation for millions of years. These endemic animals frequently interact with more recently introduced populations of non-native small mammals. From oral swab samples taken from natural populations of lemurs, rodents and shrews in the Manombo Special Reserve and surrounding area in southeastern Madagascar, we determined the complete genomes of anelloviruses from black rats (*n*=647 genomes), Webb’s tufted-tailed rats (*n*=2), mouse lemurs (*n*=4), a woolly lemur (*n*=1) and a house shrew (*n*=3). We observed distinct anellovirus lineages in the endemic lemurs and tufted-tailed rats, which we infer to be the result of their long-term geographic isolation in Madagascar. Lemur-infecting anelloviruses, in particular, do not cluster with other primate-infecting anelloviruses. In contrast, anellovirus diversity in widespread, non-native rodents (i.e. black rats) was similar to that of closely related, globally dispersed rodent species, concordant with their later introduction to Madagascar. With our large anellovirus dataset from black rats, we examined anellovirus alpha- and beta-diversity and viral co-occurrence networks. Black rat anellovirus populations showed high intra-individual variation, but the overall pool of circulating anelloviruses was consistent across age classes and sexes. Adult relative to juvenile/subadult black rats harboured richer anellovirus populations and were more connected within the co-occurrence network. Proximity between individuals and greater intra-individual viral diversity were also linked to more virus-sharing between black rats.

**Impact Statement**

While anelloviruses infecting humans have been increasingly studied due to their ubiquitous yet non-pathogenic nature, the evolutionary diversity and co-occurrence dynamics of anelloviruses in non-human animals have remained understudied. Focused on rodents and lemurs in a biodiverse landscape in southeastern Madagascar, our work contributes 657 complete anellovirus genomes that represent 10 anellovirus species, 8 of which are new species. These anelloviruses reflect the evolutionary history of their lemur and rodent hosts in Madagascar, highlighting the role of anellovirus–host codivergence in anellovirus evolution. Further, this study provides the first analysis of the effects of host and viral factors on anellovirus alpha- and beta-diversity and co-occurrence in a wildlife host population (non-native black rats). In contrast to the human-focused literature on anellovirus dynamics which have primarily employed clinical cohorts (e.g. blood-transfusion recipients, organ transplantation recipients and children with respiratory infections), we present a field-based, multi-species perspective across endemic and non-native mammals in an ecologically unique region. Collectively, this study integrates evolutionary, epidemiological, demographic and intra-host viral dynamics to provide a broader understanding of anellovirus ecology and evolution applied to non-human animals.

## Data Availability

The anellovirus genomes described in this study have been deposited in GenBank under accession numbers PX373912–PX374568. Raw read sequence data have been deposited under BioProject number PRJNA1290322, BioSample numbers SAMN49916477–SAMN49916666 and SRA numbers SRR34496596–SRR34496785. Host mitochondrial genomes identified in this study have additionally been deposited in GenBank under accession numbers PX582160–PX582284, PX591022 and PX591023.

## Introduction

Despite recent advances in determining anellovirus diversity in various animals, the demographic, co-infection, geographic and evolutionary dynamics of anelloviruses remain largely unexplored in non-human hosts. Anelloviruses are primarily mammalian- and avian-infecting viruses in the family *Anelloviridae* with circular, negative-sense ssDNA genomes [1]. Anellovirus genomes are ~1.6–3.9 kb in length, including a large, conserved *orf1* encoding the capsid protein which is usually overlapped by two to three additional shorter ORFs [2,3]. Anelloviruses are of significant interest, as they are highly prevalent animal-infecting viruses (up to ~80% prevalence in some human populations) [4] yet non-pathogenic (although inconclusively associated with a variety of diseases) [5,7]. Anelloviruses commonly co-infect their hosts, forming exceedingly diverse anellovirus populations with high inter- and intra-individual variation [8,10], and with recombination playing a key role in their evolution [3,11,15].

Oral samples have been successfully utilized to study anellovirus populations, and saliva is considered a potentially dominant route of anellovirus transmission along with proposed faecal–oral, maternal and sexual routes [1,16,21]. Anelloviruses appear to be relatively ubiquitous across mammals, with virome studies unveiling vast anellovirus diversity in hosts across taxa, from non-human primates [22,23] to rodents [24], bats [25], felids [14], dolphins [3] and marsupials [26], for example.

The island of Madagascar is home to unique, endemic mammals, including speciose non-human primate and rodent lineages. These native species interact with widespread populations of non-native animals, especially black rats [27,29]. Rodents are the most abundant, speciose mammalian order, with non-native species such as black and brown rats and house mice having extremely broad geographic ranges and displaying extraordinary ecological flexibility in their adaptation to anthropogenic landscapes. Together, these traits, combined with their importance as reservoirs of zoonotic pathogens, make rodents a compelling and appropriate system for addressing key questions in viral ecology and evolution. Known rodent anelloviruses are not monophyletic, having a complex evolutionary history; the closely related rodent-associated *Aleptorquevirus* and *Rhotorquevirus* genera are distinct from the *Wawtorquevirus* genus despite host origin overlap of Cricetidae and Muridae rodents. Some rodent anellovirus species have been found across multiple rodent host species (e.g. *Wawtorquevirus crice2* in the montane grass mouse*,* hairy-tailed bolo mouse*,* delicate vesper mouse*,* house mouse and black-footed pygmy rice rat), demonstrating that geographic overlap between rodents could promote interactions between populations of anelloviruses found in non-native and endemic Malagasy rodents.

Although primates are one of the most diverse mammalian orders, viral research in primates has been biased towards great apes – primarily humans – and Old World monkeys, neglecting entire primate lineages including gibbons, siamangs, galagos, lorises, lemurs and tarsiers. With the exception of *Chitorquevirus indri1* from an indri (lemur in Indriidae) [30], the established primate-infecting anelloviruses phylogenetically cluster together into one large primate anellovirus clade. Primate-infecting anelloviruses that have been classified in established genera have been identified from humans, chimpanzees, macaques, green monkeys, tamarins, gorillas, indri and night monkeys, showing representation across Hominidae, Aotidae, Callitrichidae, Cercopithecidae and Indriidae. For unclassified species, Lemuridae-associated anelloviruses from captive black-and-white ruffed lemurs and blue-eyed black lemurs have additionally been characterized and constitute a putative genus in a separate clade from all known primate-infecting anelloviruses including *Chitorquevirus indri1* [20].

Using oral swab samples from natural populations of primarily rodents and lemurs in the Manombo Special Reserve (MSR) and the surrounding area in southeastern Madagascar, here, we (1) expand known oral anellovirus diversity in understudied primate and rodent lineages, (2) reveal how the evolution of anelloviruses in geographically isolated, endemic species differs from widespread, introduced species in an island ecosystem and (3) investigate associations between non-native rodent host demographic factors and anellovirus diversity metrics. As part of this study, we contribute 657 anellovirus genomes, from black rats (*n*=647 genomes, *Rattus rattus*), Webb’s tufted-tailed rats (*n*=2, *Eliurus webbi*), mouse lemurs (*n*=4, *Microcebus* sp.), a woolly lemur (*n*=1; *Avahi* sp.) and a house shrew (*n*=3, *Suncus murinus*), describing their diversity. The long-term geographic isolation of endemic lemurs (~65 Mya [31]) and tufted-tailed rats (~15 Mya [32,33]) in Madagascar provides an opportunity to examine the development of distinct anellovirus lineages. In contrast, the more recent introduction of black rats (~1 kya via the Arabian trade network, though multiple invasion scenarios have been proposed [28,34]) offers a system for comparing viral diversity in a globally distributed, highly invasive species. Overall, our work uses an island landscape to illuminate anellovirus diversity and evolutionary dynamics in non-human primates and small mammals.

## Methods

### Sample collection

**Fig. 1.**
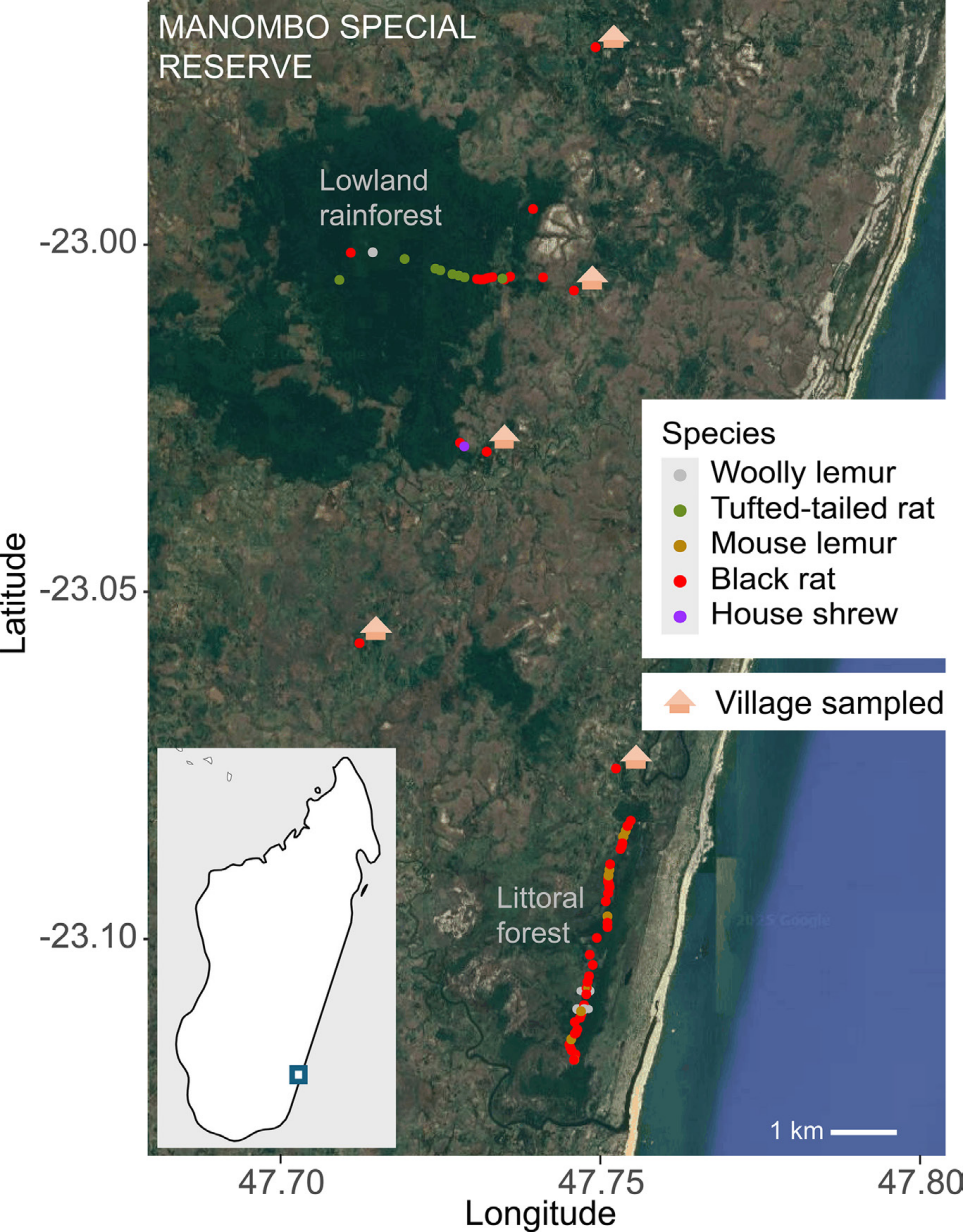
Maps of sampling points from 2022 and 2023 expeditions in the lowland rainforest, littoral forest and villages in the surrounding region of the MSR in southeastern Madagascar. House symbols highlight villages where non-native rodent sampling took place. There are many villages around both forest parcels where sampling did not take place and are therefore not depicted. The map inset shows the location of the MSR on the southeastern coast of the island of Madagascar.

The MSR in southeastern Madagascar is composed of lowland rainforest located more inland with a taller, denser canopy (termed here as the lowland rainforest parcel) along with coastal, sandy littoral forest serving as a barrier to extreme weather events such as cyclones (termed here as the littoral forest parcel). The MSR is surrounded by *ca*. 31 villages. Oral swabs were collected from animals in the MSR and surrounding area in Madagascar in October 2022 and July 2023 (Fig. 1).

Larger lemurs (black-and-white ruffed lemurs, woolly lemurs) were darted using an airgun and tranquilization with Zoletil (10–20 mg kg^−1^) [35,36]. Animals were darted only if their rump was accessible for darting and they could be safely caught with nylon mesh. Darting occurred opportunistically throughout the forested areas with an average of six darting days per forest parcel per sampling year. Mouse lemurs and rodents were trapped using 120 Sherman traps baited with banana or peanut butter set along the transects in the trees and on the ground. Sherman trapping was completed along two 4 km transects (one transect in the littoral forest, one transect in the lowland rainforest) with an average of six trap-nights at each forest parcel in each sampling season. Sherman trapping was additionally completed with one to two trap-nights in each of five villages with traps placed in homes, under homes and in cropping areas. Global positioning system (GPS) points were taken of each animal capture site. Zoletil was administered to mouse lemurs and tufted-tailed rats via syringe (10–20 mg kg^−1^) after weighing the animal. Once the animal was anesthetized, the veterinarian performed full physical health exams and monitored body temperature, heart rate and respiratory rate at the beginning and end of the exam. Age class and sex were recorded for each animal. In addition to other sample types not included in this study, oral swabs were collected with sterile flocked swabs and stored in 1 ml of Universal Transport Medium (Puritan UniTranz-RT). All larger lemurs were PIT-tagged to track individuals across multiple sampling seasons. Animal health exams and sampling lasted ~15 min per individual. Each animal was monitored by the veterinarian and, when the animal was fully mobile and alert, released within 20 m of the GPS point of the capture site.

Non-native black rats were caught using Sherman traps and euthanized via cervical dislocation or decapitation under deep anaesthesia provided by an overdose of ketamine, consistent with American Veterinary Medical Association guidelines and Madagascar National Parks recommendation. Physical health exams and sampling protocols were followed as described above. Because black rats were euthanized and therefore precluded from recapture, samples from this species are known to be from distinct individuals.

Oral swab samples were kept in a cooler on ice until being flash-frozen in liquid nitrogen at the end of the sampling day. Samples were maintained in liquid nitrogen during sample transport to Duke University (Durham, NC, USA) and stored at −80 °C until extraction.

### Nucleic acid extraction, library preparation and sequencing

DNA was extracted from 200 µl of each sample with the Roche HighPure Viral Nucleic Acid Kit to enhance viral nucleic acid representation. DNA extracts were amplified using the Illustra Templiphi rolling circle amplification kit which preferentially amplifies circular DNA. A combination of rolling circle amplified and non-amplified DNA extract (50:50) was used for library preparation. After library preparation with the Illumina DNA Prep (M) Tagmentation Kit and subsequent Illumina sequencing (150 bp paired-end reads) on the NovaSeq X Plus with Psomagen Inc., raw reads were used for viral metagenomic analysis following our established methods [20,37,39]. Host sequences were not filtered out. Paired-end reads were trimmed using Trimmomatic-0.39 [40] and *de novo* assembled with MEGAHIT v1.2.9 [41]. Circular contigs were identified based on terminal redundancy, and all contigs >1,000 nt were analysed for viral-like sequences using Diamond [42] blastx against a local viral RefSeq database (v228). Viral genomes were annotated using CenoteTaker3 v3.4.0 [43] and subsequent manual curation. A byproduct of our extraction and sequencing workflow is the identification of host mitochondrial genomes. For cases where we were able to assemble and annotate complete host mitochondrial genomes, lemur, rodent and shrew mitogenomes were deposited in GenBank under accession numbers PX582160-PX582284, PX591022 and PX591023.

### Phylogenetic analyses

Anellovirus *orf1* sequences were extracted from our dataset and from anellovirus genomes that represent species downloaded from GenBank based on the International Committee on Taxonomy of Viruses (ICTV) VMR species list [44] (VMR MSL40.v1.20250307) along with closely related sequences through blast. *Orf1* sequences were translated and aligned using mafft v7[45]. The alignment was trimmed using trimAl v1.5.1 [46] with a gap threshold of 0.2. This phylogenetic tree was inferred with IQ-Tree2 v2.4.0 [47] using the model finder option with best-fit model VT+F+R7 and rooted with members of the *Gyrovirus* genus. In addition, subtrees for those clades comprising sequences from this study were built for each clade of interest with IQ-Tree2 v2.4.0 [47] with the model finder option, and branches <80% approximate likelihood-ratio test (aLRT) support were collapsed with TreeGraph2 [48]. Best-fit models include LG+I+G4 for the *Aleptorquevirus* clade, WAG+I+G4 for the *Rhotorquevirus* clade, LG+F+I+G4 for the *Wawtorquevirus* and Webb’s tufted-tailed rat-associated clade, LG+F+G4 for the lemur-associated clade and LG+F+G4 for the shrew-associated clade.

Pairwise identity calculations between known and novel viruses were determined with the Sequence Demarcation Tool (SDT) v1.241 [49] to determine sequence similarity. For assigning genera and species for the anellovirus genomes, we used the criteria outlined by the ICTV *Anelloviridae* study group as described in Varsani *et al*. [1].

### Distribution of virus genotypes across the samples

Complete viral genomes were clustered into genotypes (for the purpose of this study) based on a 95% genome-wide pairwise identity threshold (similar to that described in Roux *et al*. [50])/genome-wide average nucleotide identity (ANI) determined using CD-Hit v4.8.1 [51]. CoverM v0.7.0 [52] was used for mapping raw reads from Illumina sequencing to a representative genome from each genotype (with -min-read-percent-identity 95%) to determine the presence of virus genotypes across all samples with a genome coverage threshold of ≥75%. The presence (≥75% genome coverage) of each genotype within a sample was counted to determine anellovirus genotype richness. SDT v1.241 [49] was additionally used to identify species-level groupings with a 69% *orf1* nucleotide pairwise identity threshold. Representatives from each of these species groups were used to visualize genome organization with Geneious.

### Recombination analysis

Species-level datasets from rodent-infecting anelloviruses were separately aligned. These alignments were used to infer recombination and break point distributions (including hotspots and cold spots) using RDP5 v5.84 [53] using default parameters and auto-masking of sequences. Recombination detection methods implemented in RDP5 v5.84 include RDP [54], GENECONV [55], BOOTSCAN [56], MAXCHI [57], CHIMAERA [58], SiScan [59] and 3Seq [60]. Events with phylogenetic support for recombination and at least three recombination detection methods with a Bonferroni-corrected *P*<0.05 were considered credible.

### Alpha- and beta-diversity metrics

To determine the associations between host age and sex and anellovirus genotype richness, diversity and population composition, alpha- and beta-diversity metrics were computed using the *vegan* package [61] in R with estimates of anellovirus genotype presence (≥75% genome coverage from CoverM v0.7.0 [52] described above) as a binary presence–absence variable. Wilcoxon (variable with 2 factors) or Kruskal–Wallis (variable with >2 factors) rank-sum tests were used to identify significant differences (*P* value <0.05) in alpha-diversity metrics (richness, Simpson’s diversity index, Shannon diversity index). Alpha-diversity metrics were highly correlated (*r*=0.60–0.91), and statistical results were consistent across richness and Shannon and Simpson’s diversity indices. We therefore report richness as the primary alpha-diversity metric. To understand differences in anellovirus population composition, we used the vegdist function in the *vegan* package [61], applying the Jaccard dissimilarity index to a matrix of anellovirus presence–absence. To assess differences in beta-diversity across age classes (juvenile/subadult, adult) and sex, we performed permutational multivariate ANOVA using the adonis2 function, with 1,000 permutations, testing whether age or sex has a significant effect (*P* value <0.05) on the dissimilarity matrix. Lastly, we conducted non-metric multidimensional scaling (NMDS) analyses using the metaMDS function in the *vegan* package [61] with Jaccard dissimilarity (1,000 iterations). The NMDS scores were plotted with age or sex group-specific ellipses to highlight overlaps in anellovirus population composition and centroids, highlighting central tendencies in ordination space.

### Intra-individual variation analysis

Using the CoverM output-derieved matrix denoting presence (≥75% genome coverage) as 1 and absence (<75% genome coverage) as 0 and the alignment of all anelloviruses characterized in this study, the degree of pairwise identity between every genome belonging to each anellovirus genotype present in each sample was computed for each black rat individual. For each black rat individual from which two or more anellovirus genotypes were identified, the degree of pairwise identity between the two most genetically different and the two least genetically different anellovirus genomes belonging to each anellovirus genotype present in each individual black rat was calculated. Intra-individual mean pairwise distance (MPD), the average pairwise distance of each genotype to each other genotype for all pairs of virus genotypes present in one black rat, was determined for each individual as a host covariate to be used in network analysis.

### Virus co-occurrence network analysis

To examine the predictors of virus sharing among black rats, a binary bipartite adjacency matrix representing interactions between black rat individuals and unique viruses was constructed. This matrix was projected into a unipartite, weighted adjacency matrix by computing the number of shared viruses between each pair of black rat individuals, using the R package *network* [62,63]. Weights represented the count of shared viruses. Next, a weighted exponential random graph model (ERGM) was fit using the ergm.count extension of the *ergm* R package [64,65]. ERGMs model how individual characteristics and dyadic patterns influence the likelihood of interactions by comparing the observed network to a distribution of random networks, accounting for non-independence inherent in network data. We used a Poisson reference distribution for the edge weights and included the sum term. To assess whether black rat characteristics predicted virus sharing, we included categorical predictors (age class and sex) using nodefactor() terms and continuous traits (body mass and intra-individual MPD, scaled to the z-score distributions) using a nodecov() term. To assess if black rat individuals were more likely to share viruses if they were the same age class or sex (i.e. to assess homophily), we included nodematch() terms for age class and sex. To investigate the effect of spatial proximity on virus sharing, we computed pairwise geographic distances of each black rat based on their GPS locations (decimal degrees) and included distances as an edge covariate. The distance between traps ranged from 0 to 16.2 km. All continuous variables were standardized. Four nodes were removed for missing characteristic values. The model was fitted using Markov chain Monte Carlo maximum likelihood estimation, with a maximum of 60 iterations, and model convergence was assessed using trace plots. We assessed collinearity using the R package *ergMargins* [66].

## Results and discussion

### Overview

**Fig. 2.**
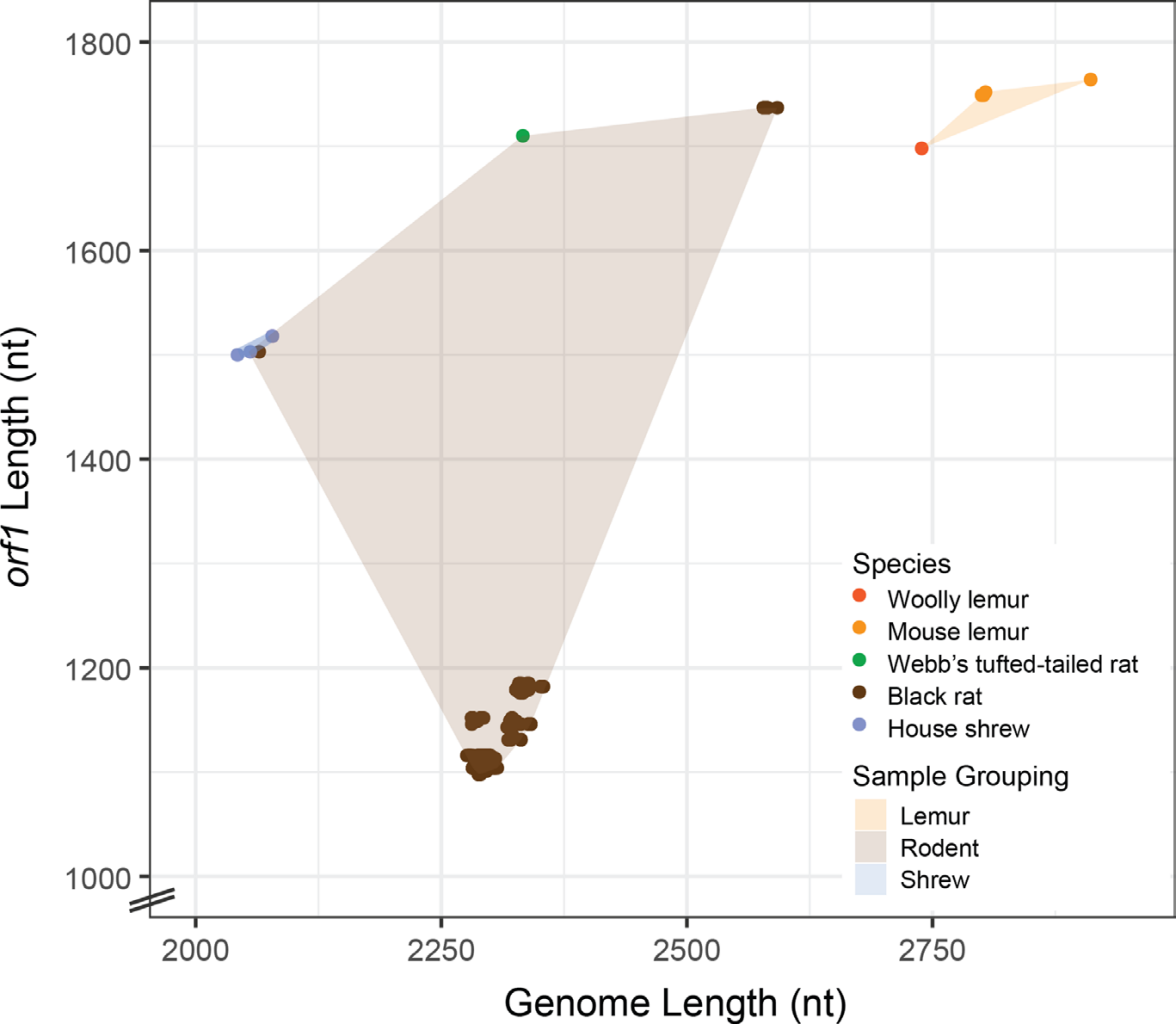
Comparison of anellovirus genome and *orf1* lengths identified from woolly lemurs, mouse lemurs, black rats, Webb’s tufted-tailed rats and a house shrew.

The oral swabs collected in this study comprise samples from 8 woolly lemurs (*Avahi* sp.)*,* 27 mouse lemurs (*Microcebus* sp.), 125 non-native black rats (*R. rattus*), 1 house shrew (*S. murinus*) and 9 Webb’s tufted-tailed rats (*E. webbi*). Complete anellovirus genomes (*n*=657) were determined from these oral swabs (Supplementary Data Sheet 1 - Data 1, available in the online Supplementary Material). All anellovirus sequences have the typical anellovirus genome organization with a large *orf1* flanked by *orf2* and *orf3* (Fig. S1). For the purpose of this study and for naming the anelloviruses, we have used a 95% pairwise identity threshold for genotypes. The 657 anellovirus genomes represent 144 genotypes. The 647 anelloviruses from black rats fall into 136 genotypes that represent 6 species (based on the 69% *orf1* pairwise identity threshold set by the ICTV *Anelloviridae* study group [1]) and have been named madamur torque teno virus 1 through 136 with the name madamur derived from **Mada**gascar **Mur**idae (Supplementary Data Sheet 1 - Data 1) [1]. Anellovirus genomes from black rats range in length from 2,054 to 2,592 nt with 44.5–55.3 mol% G+C content, while *orf1* lengths ranged from 1,098 to 1,737 nt (Figs 2 and Supplementary Data Sheet 1 - Data 1). For the rodent-infecting anelloviruses, the larger genomes are those of madamur torque teno virus 1 which are part of the genus *Wawtorquevirus* (species *Wawtorquevirus murid3* (Fig 2 and Supplementary Data Sheet 1 - Data 1). The two anelloviruses from Webb’s tufted-tailed rats represent one species and two genotypes and have been named madanes torque teno virus 1 and 2 with the name madanes derived from **Mada**gascar **Nes**omyinae. These two genomes are 2333 nt in length with an *orf1* length of 1710 nt and a G+C content of 47.5 mol% (Fig 2 and Fig. S1; Supplementary Data Sheet 1 - Data 1). The three house shrew anelloviruses, all from one individual, represent one species and three genotypes and have been named madasor torque teno virus 1 through 3, with the name madasor derived from **Mada**gascar **Sor**icidae. Anelloviruses from the house shrew are 2,039 to 2,078 nt in length with a G+C content of 44.7–48.2 mol% and have *orf1s* ranging in length from 1,500 to 1,518 nt (Fig. 2, Fig. S1 and Supplementary Data Sheet 1 - Data 1).

The four anelloviruses from mouse lemurs represent two species and two genotypes. The one anellovirus from the woolly lemur represents a single species and genotype. The three genotypes from lemurs have been named madalem torque teno virus 1 through 3, with the name madalem derived from **Mada**gascar **lem**ur. Lemur-associated anelloviruses had the largest genome sizes of those determined here, ranging from 2,739 to 2,911 nt with 48.1–50.5% GC content, while *orf1* lengths ranged from 1,698 to 1,764 nt (Fig. 2, Fig. S1).

When evaluating anellovirus species-level groupings (<69% *orf1* nucleotide pairwise identity), these 144 genotypes represent ten species, 8 of which represent new species (Supplementary Data Sheet 1 - Data 2, Fig. S1). Furthermore, of these eight new species, five represent members of three putative new genera.

Anellovirus genotype prevalence varied widely (Supplementary Data Sheet 1 - Data 4). For black rats, anellovirus genotype prevalence ranged from 0.8% (1/125 individuals; madamur TTV 15, -16, -17, -18, -23, -35 and -45) to 37.6% (47/125 individuals; madamur TTV 1). Thirty-nine anellovirus genotypes in black rats had a prevalence >10%. For mouse lemurs, anellovirus genotype prevalence ranged from 29.6% (8/27 samples; madalem TTV 2) to 40.7% (11/27 samples; madalem TTV 1). The woolly lemur anellovirus (madalem TTV 3) was present in one out of eight samples, whereas the Webb’s tufted-tailed rat anelloviruses (madanes TTV 1 and -2) were each present in one out of nine samples. Although the prevalences estimated here appear lower than values seen for some anelloviruses in humans (up to 95%) [67] and rodents (8–87%) [24], anelloviruses have generally been found to vary widely in prevalence as is seen here.

### Anellovirus diversity and phylogenetics

#### Lemur anelloviruses

**Fig. 3.**
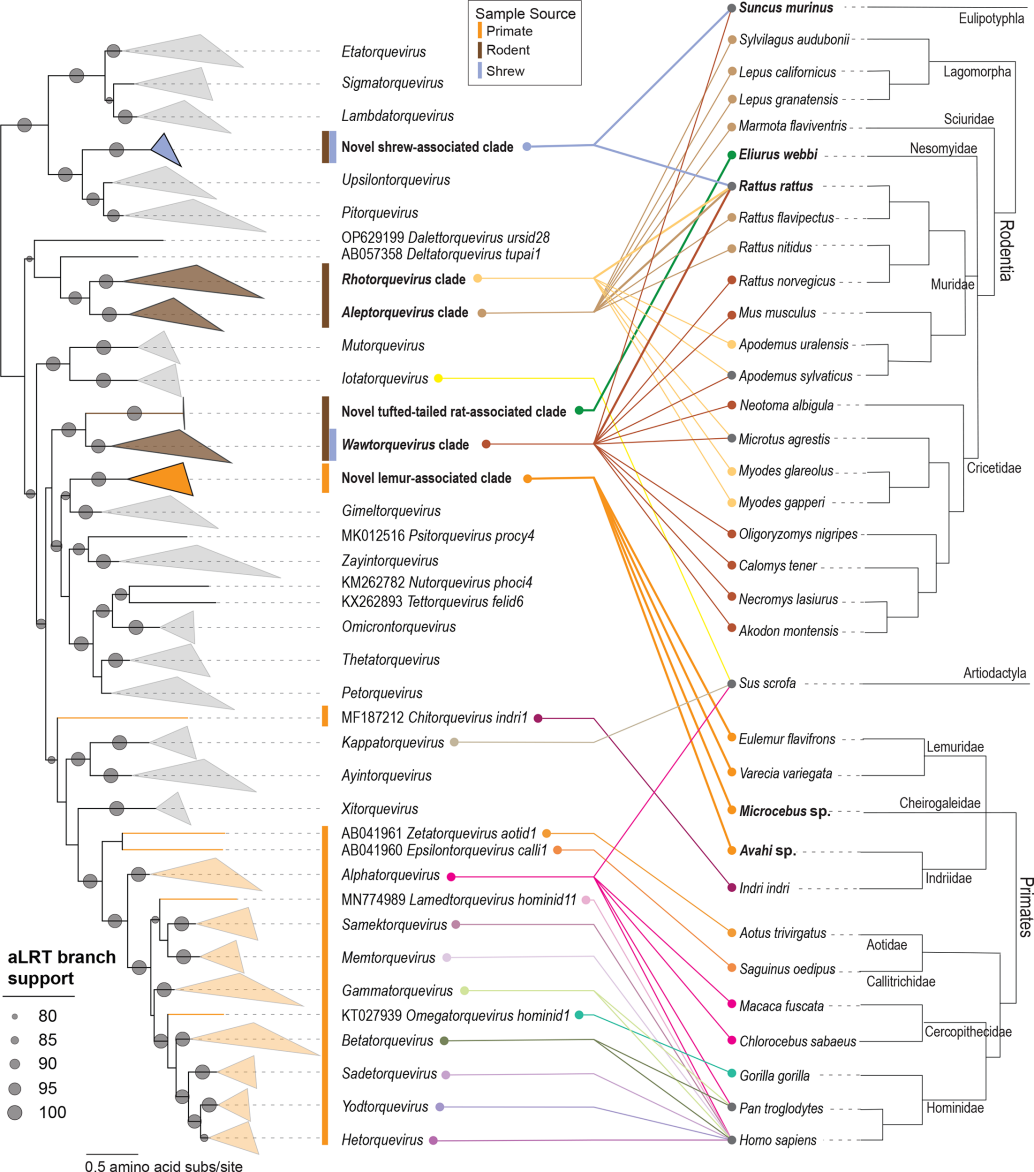
A maximum-likelihood phylogenetic tree (left) of the ORF1 amino acid sequences of anelloviruses identified in this study together with representatives of established anellovirus species. A host species cladogram of primates, rodents and shrews (right) shows associated anellovirus lineages, with each tanglegram line colour representing a different anellovirus genus. Anellovirus genera and host species investigated in this study are in bold.

**Fig. 4.**
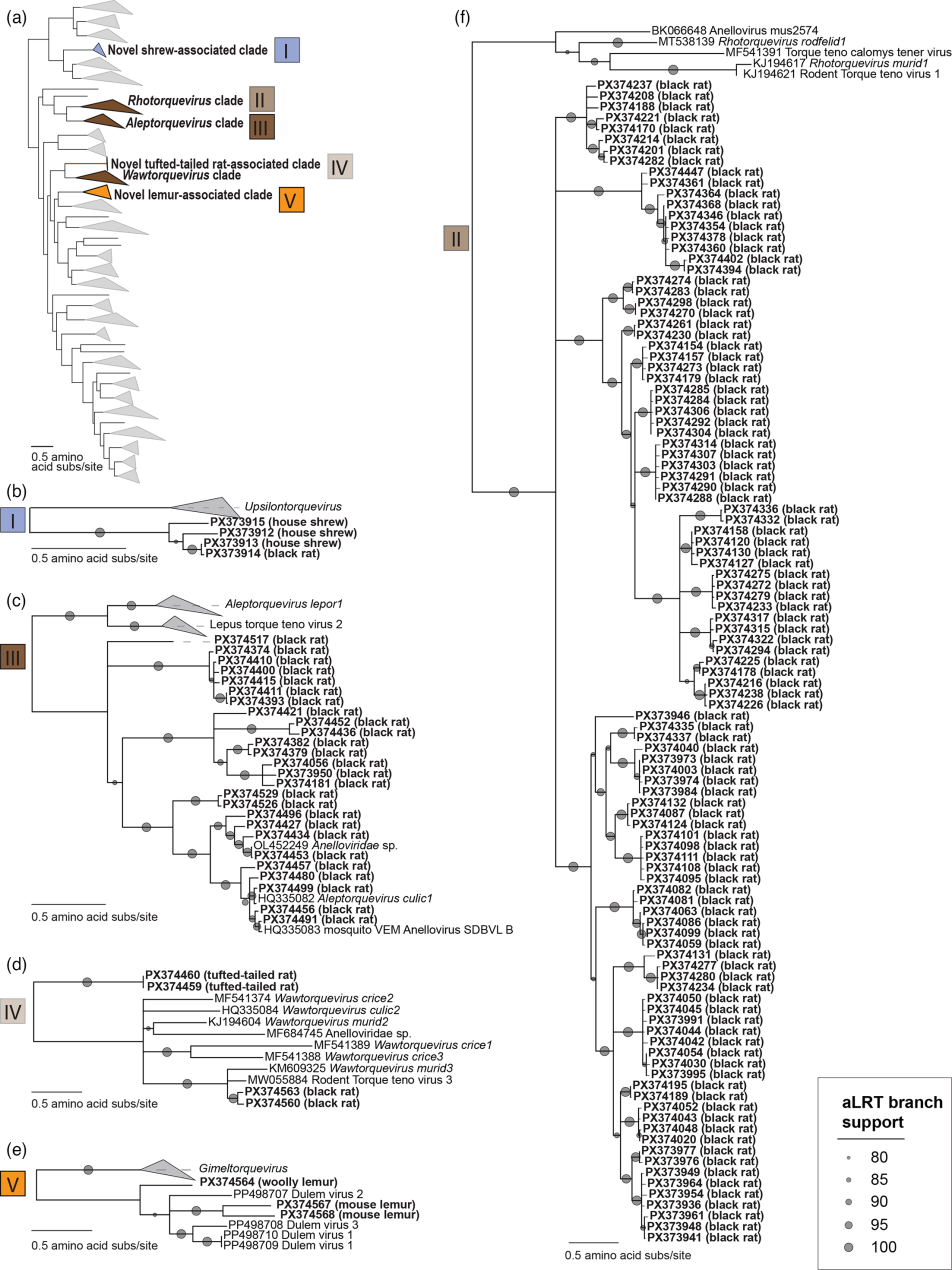
(**a**) Maximum-likelihood phylogenetic tree of the ORF1 amino acid sequences for each clade containing anellovirus sequences from this study (one representative from each genotype). Maximum-likelihood phylogenetic tree of the (**b**) novel shrew-associated anellovirus clade, (**c**) *Aleptorquevirus* clade, (**d**) Webb’s tufted-tailed rat anelloviruses and the wawtorqueviruses, (**e**) lemur-associated clade and (**f**) *Rhotorquevirus-*related clade.

Madalem TTV 1, -2 and -3 (PX374564–PX374568) genomes determined in this study share ~63% genome-wide pairwise identity with previously identified anelloviruses from oral and blood samples of captive-born lemurs in the USA [20]. From mouse lemurs, we identified four complete anellovirus genomes representing two species and three genotypes whose ORF1 amino acid sequences are phylogenetically positioned within the previously identified Lemuridae-associated lineage (Figs 3 and 4e). The three madalem TTV 1 genomes (PX374565–PX374567) share 95.7–99.1% genome-wide pairwise identity with one another and ~66% identity with the genome of madalem TTV 2 (PX374568). Madalem TTV 1 (PX374565–PX374567) *orf1* sequences share 61.3–61.6% nucleotide identity with madalem TTV 2 *orf1* (PX374568). Both madalem TTV 1 and -2 share <60% *orf1* nucleotide identity with known anellovirus sequences. Some mouse lemurs (5/27 samples) were co-infected with the two anellovirus species. Madalem TTV 3 is most similar to, with 59.3% *orf1* identity, Dulem virus 2 (PP498707) [20]. Overall, madalem TTV 1, -2 and -3 (PX374564–PX374568) share <62% *orf1* nucleotide identity with known anelloviruses and with one another. Madalem TTV 1 and -2 each represent a new anellovirus species, the first known to be characterized from Cheirogaleidae lemurs. Additionally, madalem TTV 3 (PX374564) represents a new anellovirus species, the first to be discovered from lemurs in the *Avahi* genus. Thus, building upon published lemur anelloviruses representing one established species from a wild indri [30] and three putative species from captive lemurs [20], this study contributes an additional three lemur-associated anellovirus species.

The mouse lemur anelloviruses described here represent the first anelloviruses identified from lemurs in the Cheirogaleidae family of small-bodied, nocturnal lemurs (mouse and dwarf lemurs). The *Microcebus* genus (mouse lemurs) is estimated to have diversified in the Mid-Pleistocene ~1.5 Mya [68]. The genetic divergence of mouse lemur anelloviruses expectedly corresponds with the divergence of their hosts, forming a lineage that is related to but distinct from anelloviruses found in Lemuridae and Indriidae lemurs.

Furthermore, phylogenetic analysis of ORF1 sequences shows that madalem TTV 3 (PX374564) is basal to Lemuridae- and Cheirogaleidae-associated anelloviruses in a lemur-associated lineage independent of torque teno indri virus 1 (MF187212) in the species *Chitorquevirus indri1* [30] (Figs 3 and 4e). Thus, despite the relatedness of *Indri* and *Avahi* within the Indriidae family, the anelloviruses from members of these two host genera share lower levels of similarity (~30% ORF1 amino acid identity) and form distinct phylogenetic clades (Fig. 3). The closer phylogenetic relationship of madalem TTV 3 (PX374564) to Lemuridae- and Cheirogaleidae-associated anelloviruses suggests that there are likely multiple lineages of anelloviruses circulating in Indriidae lemurs.

The woolly lemur and mouse lemur anelloviruses identified in this study are positioned with the Lemuridae-infecting anelloviruses from captive black-and-white ruffed lemurs and a blue-eyed black lemur (Fig. 4e), and collectively, they represent a new genus. This lemur-associated lineage is phylogenetically distant to the large, cohesive, primate-infecting clades (genera *Alphatorquevirus*, *Betatorquevirus*, *Epsilontorquevirus*, *Gammatorquevirus*, *Hetorquevirus*, *Memtorquevirus*, *Omegatorquevirus*, *Sadetorquevirus*, *Samektorquevirus*, *Yodtorquevirus* and *Zetatorquevirus*) of New World monkey, Old World monkey and ape anelloviruses (Fig. 3). *Hetorquevirus*, *Lamedtorquevirus*, *Memtorquevirus*, *Samektorquevirus*, *Sadetorquevirus* and *Yodtorquevirus* have thus far only been characterized in humans. Yet, viruses in *Alphatorquevirus* have been characterized in humans, chimpanzees, macaques and green monkeys, along with two sequences from pigs. Viruses in *Gammatorquevirus* and *Betatorquevirus* are human- and chimpanzee-associated. Epsilontorqueviruses have been identified in cotton-top tamarins, omegatorqueviruses in gorillas and zetatorqueviruses in night monkeys. The monkey- and ape-infecting anellovirus lineage has a closer phylogenetic relationship with pig (*Kappatorquevirus*) and opossum (*Xitorquevirus*) anelloviruses than with lemur anelloviruses (Fig. 3). These results build on our previous findings in captive lemurs and provide preliminary support for our overarching hypothesis that lemur-associated anelloviruses (from both captive and wild lemurs) are divergent from other primate-associated anelloviruses, likely due to the geographic isolation and speciation of lemurs in Madagascar. Because lemurs diversified over millions of years into more than 100 species across the diverse ecosystems of Madagascar, they form a natural experiment in host–virus evolution with our phylogenetic results providing initial evidence of a broader model of host–anellovirus co-divergence in ancient primate lineages as viral diversity reflects deep host speciation. Additionally, we highlight that while very few of the viruses that infect strepsirrhines (lemurs, galagos, pottos and lorises) have been characterized, strepsirrhines may have particularly distinct anelloviruses with the potential to vastly expand what we know about primate anellovirus diversity.

#### Webb’s tufted-tailed rat anelloviruses

Two anellovirus genomes, madanes TTV 1 and -2 (PX374459 and PX374460), which share 93.7% genome-wide pairwise identity, were identified in endemic Webb’s tufted-tailed rat oral swab samples. The ORF1 sequences form a new clade phylogenetically related to those in the genus *Wawtorquevirus*, which primarily have been found in rodents. Anelloviruses classified in *Wawtorquevirus* include those identified from a striped field mouse, montane grass mouse, hairy-tailed bolo mouse, delicate vesper mouse, black-footed pygmy rice rat, short-tailed field vole, white-throated woodrat, wood mouse, house mouse and brown rat (Fig. 3). The *orf1* sequences of madanes TTV 1 and 2 share <59% pairwise nucleotide and 34% amino acid identity with members of the *Wawtorquevirus* genus. Although based on a limited sample size of two genomes, given this low degree of similarity to members of *Wawtorquevirus*, Webb’s tufted-tailed rat anelloviruses form a distinct lineage representing a new species and could be members of a potential new genus of the *Anelloviridae* family (Supplementary Data Sheet 1 - Data 2). The endemic rodent anelloviruses identified here were not present in the samples of any other rodent species investigated in this study. Given that they represent a unique rodent lineage, this supports a long co-evolutionary history between Malagasy tufted-tailed rats that is likely attributable to their long-term geographic isolation. These are the first anellovirus genomes from a rodent in the Nesomyidae family.

#### Non-native shrew anelloviruses

Three anellovirus genomes, madasor TTV 1, -2 and -3 (PX373912, PX373913 and PX373915), were identified from an Asian house shrew oral swab sample collected at the edge of the lowland rainforest. These genomes represent a new species within a putative new genus (Supplementary Data Sheet 1 - Data 2) in the family *Anelloviridae* and share <61.8% *orf1* nucleotide and 39.9% amino acid identity with members of the *Upsilontorquevirus* genus (Fig. 4). The only other anellovirus previously identified from shrews is the partial genome of rodent TTV 3 (MW055879; China) [69], a member of the species *Wawtorquevirus crice2* (Fig. 4). Furthermore, madamur TTV 18 (PX373914) shares 98.0% genome-wide nucleotide identity with one of the Asian house shrew anellovirus genomes, madasor TTV 3 (PX373913). As this black rat (MNB22_090) and Asian house shrew (MNB22_100) were captured in the same habitat at the edge of the lowland rainforest, it may be that the black rat came into close contact with a house shrew or their associated faecal matter, possibly even as a food source, since rodents engage in coprophagy [70]. This finding may represent co-exposure or rare cross-host transfer between two non-native species in the MSR ecosystem. Although positioned most closely to *Upsilontorquevirus* and *Pitorquevirus*, the shrew-derived anelloviruses described here potentially form a putative new genus (Fig. 3).

These shrew anelloviruses represent some of the first complete anellovirus genomes from an animal in the Eulipotyphla order (hedgehogs, solenodons, moles and ‘true’ shrews). Asian house shrews are estimated to have been introduced to Madagascar around 0.7–1 kya [71]. Given the scarcity of anellovirus sequences for Eulipotyphla, more anellovirus data from this group are needed to determine if these non-native shrews in Madagascar harbour similar anellovirus populations to shrews in other geographic regions.

#### Non-native black rat anelloviruses

We identified 646 complete anellovirus genomes from black rats that span rodent-infecting lineages (madamur TTV 1–136; PX373914, PX373916–PX374458 and PX374461–PX374563): 388 were identified from black rats in the littoral forest, 134 from black rats in the lowland forest and 124 from black rats in villages. Complete genomes of madamur TTVs from this study can be assigned to the established genera *Aleptorquevirus* (*n*=26 genotypes; 189 genomes), *Rhotorquevirus* (*n*=107 genotypes; 423 genomes) and *Wawtorquevirus* (*n*=2 genotypes; 34 genomes) (Supplementary Data Sheet 1 - Data 2; Figs 3 and 4). Viruses in *Aleptorquevirus* have previously been identified from rodents (Tanezumi rat, Himalayan field rat and yellow-bellied marmot), lagomorphs (Granada hare, black-tailed jackrabbit and desert cottontail) and mosquitoes – likely from a recent mammal bloodmeal. Viruses in *Rhotorquevirus* and *Wawtorquevirus* have been primarily identified from members of the Muridae (wood mouse, ural field mouse) and Cricetidae (short-tailed field vole, bank vole) families. The anelloviruses from this study therefore expand known diversity, hosts and geographic ranges for *Aleptorquevirus*, *Rhotorquevirus* and *Wawtorquevirus*.

Working from the 69% *orf1* nucleotide identity species demarcation threshold for the family *Anelloviridae* [1], the anellovirus genomes from black rats represent eight tentative black rat-associated species (Fig. S1; Supplementary Data Sheet 1 - Data 2). Genomes of madamur TTV 1 (*n*=26) and madamur TTV 55 (*n*=8) are all members of the genus *Wawtorquevirus* sharing >73.5% *orf1* nucleotide identity with other members of the species *Wawtorquevirus murid3*. Of 189 genomes that are part of the genus *Aleptorquevirus*, 73 are members of the species *Aleptorquevirus culic1* and represent 11 genotypes (madamur TTV 14, -21, -62, -65, -81, -83, -92, -97, -101, -102 and -130). Fifty-three genomes that span 8 genotypes (madamur TTV 51, -54, -56, -93, -94, -96, -110 and -129) represent a new species and 63 genomes that span 7 genotypes (madamur TTV 2, -32, -57, -77, -78, -99 and -105) represent a second new species (Supplementary Data Sheet 1 - Data 2). As there are currently two established *Aleptorquevirus* species, this study adds an additional two putative species to the *Aleptorquevirus* genus. A large number of genomes (*n*=423; spanning 107 genotypes) are members of the *Rhotorquevirus* genus; none can be assigned to currently established species and these represent a single new species (Supplementary Data Sheet 1 - Data 2). This new species expands *Rhotorquevirus* diversity as the *Rhotorquevirus* genus currently has only two established species.

Our findings reveal that while black rat anelloviruses have rich populations with unappreciated degrees of divergence, all are positioned within known rodent-infecting lineages and demonstrate lower divergence from known lineages than endemic lemur and rodent anelloviruses. These similarities are expected given the global distribution of black rats and their relatively recent introduction by humans to Madagascar ~1–3 kya, likely via the Arabian trade network in the Indian Ocean. Black rats in Madagascar tend to be most closely related to those of the Arabian Peninsula, and there have likely been two main colonization events via boats, although other post-colonization migration events are possible [28,34].

### Anellovirus recombination

**Fig. 5.**
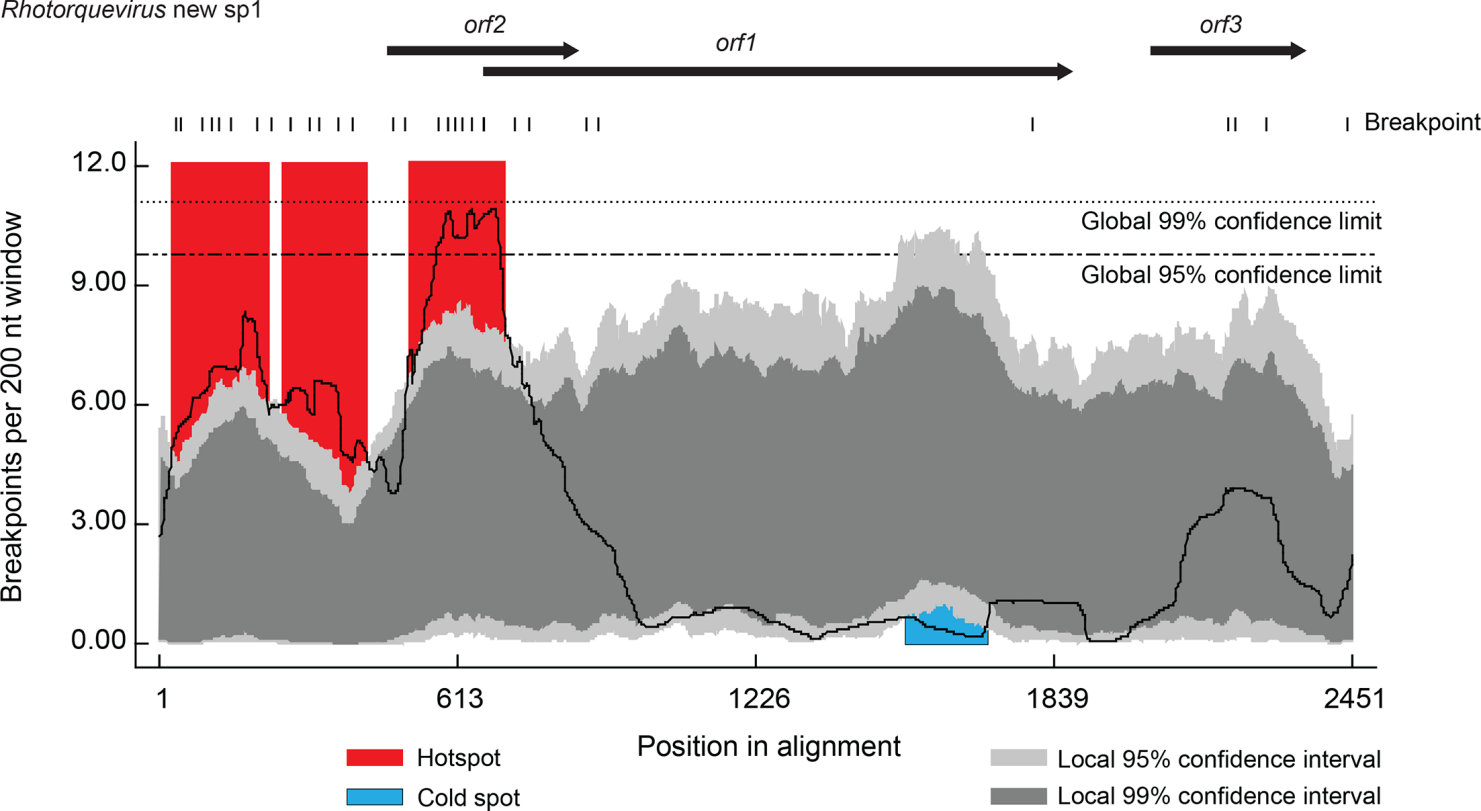
Plot of the distribution of the recombination break points of *Rhotorquevirus* new sp 1 group with recombination hotspots indicated with a red background and cold spots indicated with a blue background. 95 and 99% confidence interval plots are shown in light and dark grey, respectively.

Recombination and the underlying mechanisms during replication in ssDNA viruses are reviewed in Martin *et al*. [72] and would require co-infection of the same cell with homologous viruses. Recombination has been identified as one of the primary drivers promoting anellovirus diversity, contributing to the development of new anellovirus species and genera, in both humans and non-human animals [3,11, 12, 15, 72]. However, in the case of rodent-infecting anelloviruses, prior to this study, there have been no datasets large enough to determine recombination patterns. In our dataset, of the ten species-level rodent anellovirus groupings, we identified evidence of recombination in six of these (Supplementary Data Sheet 1 - Data 3). The highest number of recombination events (*n*=24) was found in members of the new *Rhotorquevirus* species (423 members). For this group, the recombinant regions ranged from 113 to 905 nt with recombination hotspots in the non-coding region and in the region spanning the 5′ end of *orf*1 and the central portion of *orf*2 and a cold spot near the 3′ end of *orf*1 (Fig. 5). Previously, recombination hotspots have also been found in the non-coding regions of other anelloviruses [1,3,12]. Although the hotspot in the genomic region spanning the 5′ end of *orf*1 and the central portion of *orf*2 of the new *Rhotorquevirus* species is unusual, a recombination hotspot spanning a similar region has been identified for members of the genus *Lambdatorquevirus phoci5* (Torque teno pinniped virus 8) that have been found to be associated with Weddell seals [2].

For the members of the species *Aleptorquevirus culic1* (*n*=78), we identified two recombination events (622 to 895 nt recombinant regions), for *Aleptorquevirus* new sp1 (*n*=53) one event (143 nt recombinant region), *Aleptorquevirus* new sp2 (*n*=63) four events (301 to 917 nt recombinant regions), *Wawtorquevirus murid3* (*n*=36) five events (397 to 820 nt recombinant regions) and for new genus 1 new sp1 (*n*=4) one event (34 nt recombinant region) (Supplementary Data Sheet 1 - Data 3). In general, recombination is prevalent in these genomes and provides evidence that co-infections are contributing to the generation of new anellovirus lineages in rodents.

### Richness, population composition and intra-individual variation of anelloviruses in black rats

Given the large populations of black rats in Madagascar, it is not surprising that this species was trapped most frequently, yielding our large sample set and allowing for demographic analyses. Because black rats were euthanized, each sample represents a distinct individual. Young juvenile or subadult (*n*=63, 3 weeks to 5 months old), hereafter referred to as young, and adult (*n*=53, >5 months old) black rats were trapped in the littoral forest and lowland rainforest of MSR and in five villages varying in distance from the forest parcels. It should be noted that rats in the ‘young’ category had already left their nests and were roaming independently through these sampling spaces. Fifty-eight of the black rats were females and 60 were males. For two individuals, we were unable to assess age class and we therefore removed these from the age-partitioned analyses.

In humans, intra-individual anellovirus populations differ by age and gender, with both the diversity of populations and viral loads increasing with age and tending to be highest in males [11,74, 75]. Despite large inter-individual variation in anellovirus populations, the structure of intra-individual anellovirus populations appears to be relatively stable [76]. The impact of host demographic factors on anellovirus species richness, population composition and inter-host-individual similarities and differences has not yet been tested in non-human animal populations.

**Fig. 6.**
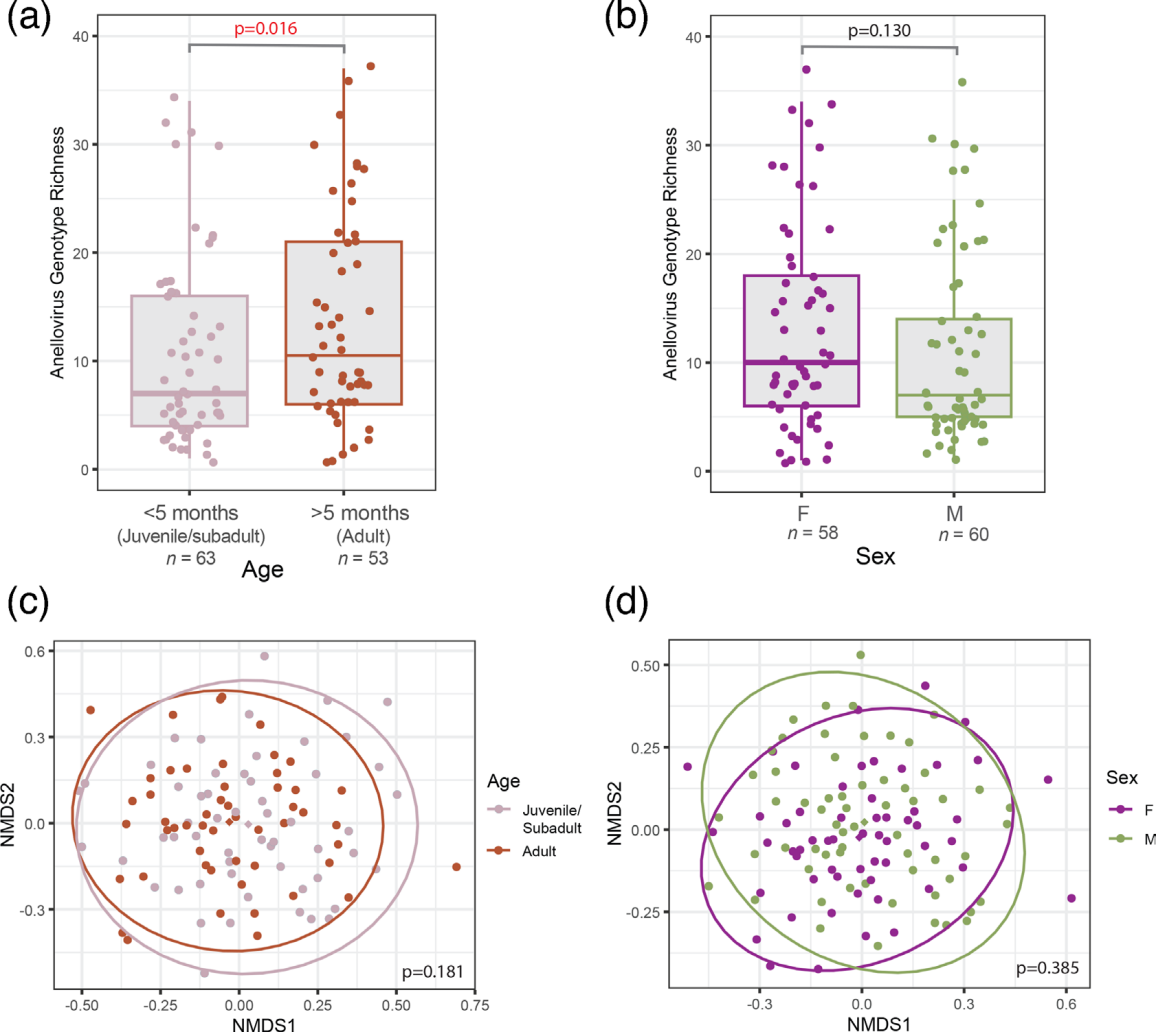
Anellovirus genotype richness in black rats showing the effects of (**a**) age class and (**b**) sex. Summary of the NMDS showing differences in intra-individual anellovirus population composition (quantified using beta-diversity scores) by (**c**) age class and (**d**) sex.

We examined whether the age class and sex of black rats have any discernible effects on measures of intra-host anellovirus genotype richness, the number of distinct genotypes found infecting each individual rat. Comparing this alpha-diversity metric for 63 young rats to those for 53 adult rats revealed a moderate association (*P*=0.016; Wilcoxon rank sum test) between age and intra-individual genotype richness. Specifically, young black rats had significantly lower anellovirus genotype richness than adults, a pattern that conforms with observations in humans (Fig. 6a) [11]. It is unclear what drives this association in either humans or rats, but it is plausible that, as an individual ages, they are repeatedly exposed to anelloviruses within their environment and become infected with a progressively increasing array of anellovirus genotypes throughout their lifespan. Comparing the alpha-diversity metric for 60 male rats to 58 female rats revealed no association between intra-individual genotype richness and sex (*P*=0.162; Wilcoxon rank sum test) (Fig. 6b).

Using the beta-diversity metric, we were unable to discern any impacts of age or sex on differences in anellovirus population compositions between pairs of individuals (Fig. 6c, d). For diversity analyses, we further stratified our dataset by age class and sex to ensure there were no observed sex effects that may have been confined to a specific age class, but we found no significant associations. This lack of any clear differences in intra-individual anellovirus diversity between age classes and sexes implies that different age classes and sexes may be exposed to the same populations of anelloviruses. While the types and lengths of social or environmental interactions that foster anellovirus genotype sharing may differ across age or sex, black rats sampled in this study likely generally have access to similar habitats, resources and interactions with conspecifics throughout forest and village areas.

**Fig. 7.**
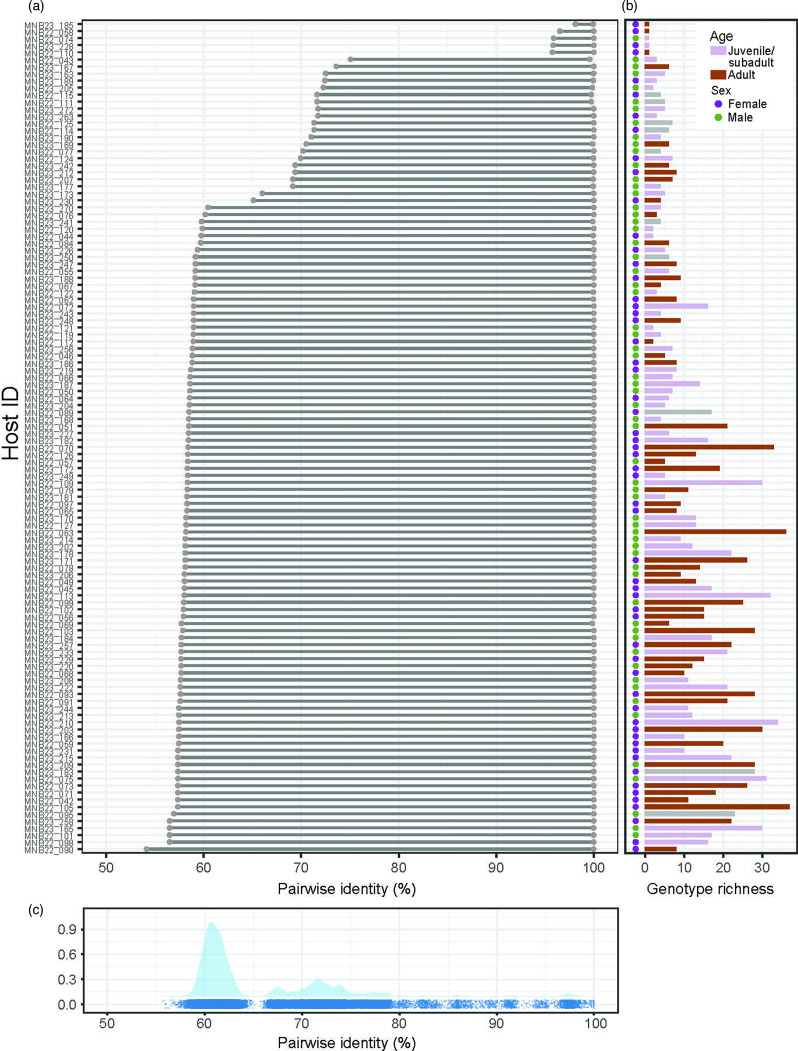
(**a**) Intra-individual anellovirus diversity range in black rat individuals, ordered from the lowest to the highest degree of pairwise identity between the two most genetically different anellovirus genomes sampled from each individual black rat from which two or more anellovirus genotypes were sampled. (**b**) Summary of the number of genotypes present in each individual black rat is shown in the bar plot with bars coloured by age class and circles next to each bar depicting sex. (**c**) The distribution of pairwise identities of the *orf*1 gene across all the anelloviruses identified from rodents in this study.

Further, intra-individual variation in anellovirus genotype richness and population composition may be overshadowing the age or sex differences that might be revealed by alpha- or beta-diversity metrics. Indeed, the black rats in this study demonstrate high variations in intra-individual anellovirus populations (lower pairwise identity) at the genotype level (Fig. 7a, c). Anellovirus genotype richness in individual black rats ranged from 0 to 37 genotypes, and 46% of black rats were co-infected with >9 genotypes (Supplementary Data Sheet 1 - Data 5). MPD for individual black rats ranged from 0 to 6.38. When we examined the effects of age and sex on intra-individual differences in pairwise similarity, no significant effects were seen (*P*_age_=0.27, *P*_sex_=0.15; Wilcoxon rank sum test).

As the alpha-diversity, beta-diversity and differences in pairwise similarity metrics were not detectably impacted by sex, we must conclude that, unlike trends seen in humans, behavioural, hormonal or immunological differences between male and female black rats do not appear to impact the diversity of anellovirus populations found infecting them. Our findings do, however, show that, at the scale of anellovirus genotype richness at least, individual black rats accumulate anelloviruses throughout their lifespans from, presumably, a consistently diverse pool of ubiquitous anelloviruses found in the environments where they live and the other animals with which they interact.

### Anellovirus genotype co-occurrence network in black rats

**Fig. 8.**
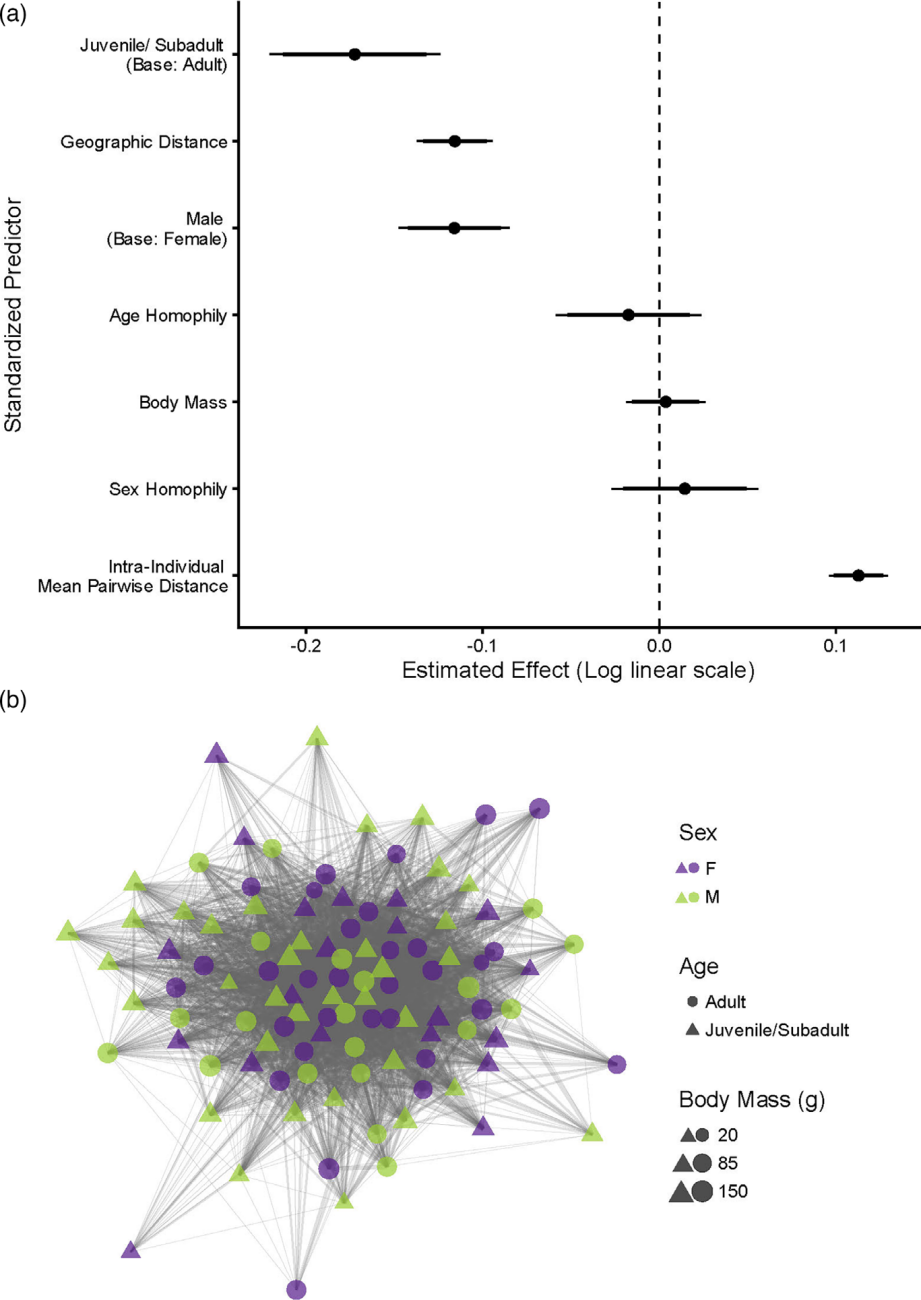
(**a**) ERGM coefficient plot showing black rat traits associated with virus sharing. Thick bars represent 90% confidence intervals, and thin bars represent 95% confidence intervals. Note that effect sizes are on the log-linear scale (i.e., for every 1 unit change in a given variable, the number of shared viruses changes by e^b, where b is the estimated effect size). Negative coefficients represent that a given variable is associated with fewer shared viruses; positive coefficients represent that a given variable is associated with more shared viruses. (**b**) Network representing black rat individuals (nodes) and the viruses they share with other black rat individuals (edges). Edges are weighted by the number of shared viruses.

To determine the factors influencing anellovirus genotype co-occurrence among black rats, we tested the predictive effects of host (node) covariates (age class, sex, geographic distance, body mass and intra-individual MPD) on host centrality to the viral genotype co-occurrence networks (Fig. 8a, b). An ERGM was chosen for this dataset given that the probability of viral genotype co-occurrence is not independent to each pair of nodes; rather, it results from a generative process that is shaped by a broader ecological context. Unlike traditional regression approaches which assume that pairs of nodes (i.e. dyads) are independent, ERGMs account for the dependencies between nodes (i.e. rat individuals). We consider dependencies of rat traits, homophily and spatial clustering.

For age class, young black rat (<5 months old) individuals were estimated to have 16% fewer shared viruses than adult black rats (estimate=−0.172, se=0.025, *P*<0.001; Fig. 8a). Adults were more connected in the network, consistent with our finding of higher anellovirus genotype richness in adults, likely due to cumulative exposure to anelloviruses across time. This suggests that adult black rats have experienced more virus sharing with conspecifics or the environment over time compared to younger rats. Compared to females, males shared 11% fewer viruses with other black rats (estimate=−0.116, se=0.016, *P*<0.001; Fig. 8a). This sex effect could be attributable to a variety of factors such as higher frequencies of female interactions with offspring (e.g. vertical or horizontal transmission of viruses from mothers to babies during birth, breastfeeding and grooming [77]), behavioural differences (e.g. dominance hierarchies and time in nest [78]) or spatial distributions (e.g. differences in home ranges between males and females [79]). Providing support that these age or sex effects are not due to adult-adult interactions or female–female interactions, we did not detect homophily for age class (estimate=−0.017, se=0.021, *P*=0.407; Fig. 8a) or sex (estimate=0.014, 0.021, *P*=0.496; Fig. 8a). In other words, black rats were estimated to share a similar number of viruses with individuals in their same age class compared to the other age class and with individuals of the same sex compared to the opposite sex.

There was also no detectable association between degrees of virus co-occurrence and body mass (estimate=0.004, se=0.012, *P*=0.750; Fig. 8a). Although loss of body mass can be associated with poor health, this finding highlights that rats with lower body masses do not have more virus-sharing interactions. Unhealthy rats may not share more anelloviruses than healthy rats, underlining the non-pathogenic nature of anelloviruses.

Black rats with greater intra-individual viral diversity shared more viruses with other black rats (estimate=0.113, sd=0.009, *P*<0.001; Fig. 8a). Thus, black rat individuals with more diverse viral populations were more strongly connected within the co-occurrence network, serving as key nodes contributing to the maintenance of this diverse anellovirus pool.

Geographic distance between the sites where pairs of rats were sampled (trap-to-trap distance) was negatively associated with degrees of virus sharing between the pairs of rats (estimate=−0.116, se=0.011, *P*<0.001; Fig. 8a). As expected, black rats shared more viruses with individuals who were in closer spatial proximity. More proximate individuals are likely to have more home range overlap, travel through the same spaces and interact with similar conspecifics. Overall, age class, sex, geographic proximity and intra-individual MPD may help predict degrees of virus co-occurrence among black rats. These findings suggest that anellovirus co-occurrence between black rats at the MSR is structured across time (with adults having high connectivity in the network and richer anellovirus populations) and space (as rats closer in proximity have more virus-sharing interactions). Moreover, within-host patterns (high intra-individual MPD) and social interactions (such as mother–offspring interactions) plausibly also drive co-occurrence dynamics. These results represent one of the first applications of virus genotype-level network modelling for a DNA virus system in a natural animal population. Further, while modelling approaches have primarily been applied to acute pathogens, this work forms a foundation for examining ecological and evolutionary dynamics for chronic, non-pathogenic viral populations exhibiting frequent co-infection.

## Conclusions

Lemur-associated anelloviruses characterized in this study from mouse lemurs (Cheirogaleidae) and woolly lemurs (Indriidae) were phylogenetically most closely related to captive-born Lemuridae-associated anelloviruses and together form a putative new genus. Within this lemur-derived anellovirus lineage, we see diverse anelloviruses across the represented lemur families (Cheirogaleidae, Indriidae and Lemuridae), a pattern consistent with virus–host co-evolution. As all other primate-associated (Hominidae, Aotidae, Callitrichidae and Cercopithecidae) anelloviruses fall into one large, separate clade, the geographic isolation and subsequent diversification of lemurs are reflected in the phylogenetic relationships between the lemur-associated anelloviruses. Further, endemic Webb’s tufted-tailed rat anelloviruses represent a putative new genus positioned near *Wawtorquevirus* and display more divergence than anelloviruses identified in black rats. Given the relatively recent introduction of black rats to Madagascar, anelloviruses from this widespread host species were all positioned within the known rodent-infecting lineages of *Aleptorquevirus*, *Rhotorquevirus* and *Wawtorquevirus*. The black rat *Rhotorquevirus* dataset was particularly large, including 107 different genotypes representing 1 new species in a genus with only two existing established species. Through this study, black rats are the first rodent species in which anelloviruses across the three major rodent-infecting anellovirus genera have been identified. Focused on a unique ecological region, this study highlights how endemic mammalian hosts (i.e. lemurs and tufted-tailed rats) harbour deeply divergent anellovirus lineages, while non-native mammalian hosts (i.e. black rats) retain globally consistent viral signatures. Phylogenetic analyses point towards a co-evolutionary hypothesis; however, the sampling of more host taxa and the characterization of more anellovirus genomes will help to resolve this, while potentially revealing other signals such as host-restricted transmission, ecological filtering, sampling structure or vector transmission. Broadly, non-pathogenic, persistent viruses that are ubiquitous in mammals may serve as valuable markers of mammalian evolutionary history, population structure and biogeography.

Using our dataset of 646 black rat-associated anellovirus genomes across 118 individuals, we examined the predictive effects of demographic factors on anellovirus alpha- and beta-diversity metrics and virus-sharing interactions, tests that have previously been carried out only for human-infecting anelloviruses. As in human anelloviruses, black rat anellovirus populations demonstrated high degrees of intra-individual diversity, although the pool of anelloviruses circulating in this population was consistent across age classes and sexes. We found that individual adult (>5 months) black rats tend to host richer anellovirus populations that are more connected within the viral genotype co-occurrence network than those hosted by juvenile/subadults. Sex did not have a significant effect on genotype richness, composition or diversity of anellovirus populations found within individual black rats, inconsistent with findings in humans that sex-associated behavioural, hormonal or immunological differences contribute to greater intra-individual anellovirus richness and diversity in males than in females. Despite this, anellovirus populations in females were found to be more connected in the virus genotype co-occurrence network. We additionally found that black rats in closer proximity to one another and hosting anelloviruses with higher degrees of diversity (estimated by higher intra-individual MPD) shared more viruses. This work serves as the first analysis of the significance of demographic factors on anellovirus dynamics in a natural animal population (black rats) and reveals the diversity of anelloviruses infecting endemic and non-native mammals with rich evolutionary histories living together in Madagascar. Collectively, we find concordant evolutionary, epidemiological, demographic and intra-host viral dynamics seen in other anellovirus–host associations and further evidence that anellovirus–host co-divergence is a dominant theme in anellovirus evolution.

## Supplementary material

10.1099/mgen.0.001681 Supplementary Data Sheet 1.

10.1099/mgen.0.001681 Fig. S1.

## Abbreviations

ERGM: exponential random graph model
GPS: global positioning system
ICTV: International Committee on Taxonomy of Viruses
MPD: mean pairwise distance
MSR: Manombo Special Reserve
NMDS: non-metric multidimensional scaling
SDT: Sequence Demarcation Tool
TTV: torque teno virus

## References

[1] Varsani A, Kraberger S, Opriessnig T, Maggi F, Celer V (2023). Anelloviridae taxonomy update 2023. Arch Virol.

[2] Butkovic A, Kraberger S, Smeele Z, Martin DP, Schmidlin K (2023). Evolution of anelloviruses from a circovirus-like ancestor through gradual augmentation of the jelly-roll capsid protein. Virus Evol.

[3] De Koch MD, Krupovic M, Fielding R, Smith K, Schiavone K (2025). Novel lineage of anelloviruses with large genomes identified in dolphins. J Virol.

[4] Taylor LJ, Keeler EL, Bushman FD, Collman RG (2022). The enigmatic roles of Anelloviridae and Redondoviridae in humans. Curr Opin Virol.

[5] Biagini P, Charrel RN, de Micco P, de Lamballerie X (2003). Association of TT virus primary infection with rhinitis in a newborn. Clin Infect Dis.

[6] Pan S, Yu T, Wang Y, Lu R, Wang H (2018). Identification of a torque teno mini virus (TTMV) in hodgkin’s lymphoma patients. Front Microbiol.

[7] Al-Qahtani AA, Alabsi ES, AbuOdeh R, Thalib L, El Zowalaty ME (2016). Prevalence of anelloviruses (TTV, TTMDV, and TTMV) in healthy blood donors and in patients infected with HBV or HCV in Qatar. Virol J.

[8] Okamoto H, Takahashi M, Nishizawa T, Ukita M, Fukuda M (1999). Marked genomic heterogeneity and frequent mixed infection of TT virus demonstrated by PCR with primers from coding and noncoding regions. Virology.

[9] Sabbaghian M, Gheitasi H, Shekarchi AA, Tavakoli A, Poortahmasebi V (2024). The mysterious anelloviruses: investigating its role in human diseases. BMC Microbiol.

[10] Spandole S, Cimponeriu D, Berca LM, Mihăescu G (2015). Human anelloviruses: an update of molecular, epidemiological and clinical aspects. Arch Virol.

[11] Cebriá-Mendoza M, Beamud B, Andreu-Moreno I, Arbona C, Larrea L (2023). Human Anelloviruses: influence of demographic factors, recombination, and worldwide diversity. Microbiol Spectr.

[12] Arze CA, Springer S, Dudas G, Patel S, Bhattacharyya A (2021). Global genome analysis reveals a vast and dynamic anellovirus landscape within the human virome. Cell Host Microbe.

[13] Fahsbender E, Burns JM, Kim S, Kraberger S, Frankfurter G (2017). Diverse and highly recombinant anelloviruses associated with Weddell seals in Antarctica. Virus Evol.

[14] Kraberger S, Serieys LEK, Richet C, Fountain-Jones NM, Baele G (2021). Complex evolutionary history of felid anelloviruses. Virology.

[15] De Koch MD, Sweeney N, Taylor JE, Lucas F, Ratheesh NK (2025). Diverse anelloviruses identified in leporids from arizona (USA). Viruses.

[16] Deng X, Terunuma H, Handema R, Sakamoto M, Kitamura T (2000). Higher prevalence and viral load of TT virus in saliva than in the corresponding serum: another possible transmission route and replication site of TT virus. J Med Virol.

[17] Goto K, Sugiyama K, Ando T, Mizutani F, Terabe K (2000). Detection rates of TT virus DNA in serum of umbilical cord blood, breast milk and saliva. Tohoku J Exp Med.

[18] Inami T, Konomi N, Arakawa Y, Abe K (2000). High prevalence of TT virus DNA in human saliva and semen. J Clin Microbiol.

[19] Kaczorowska J, van der Hoek L (2020). Human anelloviruses: diverse, omnipresent and commensal members of the virome. FEMS Microbiol Rev.

[20] Paietta EN, Kraberger S, Lund MC, Vargas KL, Custer JM (2024). Diverse circular DNA viral communities in blood, oral, and fecal samples of captive lemurs. Viruses.

[21] Crane A, Goebel ME, Kraberger S, Stone AC, Varsani A (2018). Novel anelloviruses identified in buccal swabs of Antarctic fur seals. Virus Genes.

[22] Cosentino MAC, D’arc M, Moreira FRR, Cavalcante LT de F, Mouta R (2022). Discovery of two novel torque teno viruses in *Callithrix penicillata* provides insights on Anelloviridae diversification dynamics. Front Microbiol.

[23] Hrazdilová K, Slaninková E, Brožová K, Modrý D, Vodička R (2016). New species of torque teno miniviruses infecting gorillas and chimpanzees. Virology.

[24] Nishiyama S, Dutia BM, Stewart JP, Meredith AL, Shaw DJ (2014). Identification of novel anelloviruses with broad diversity in UK rodents. J Gen Virol.

[25] Bolatti EM, Viarengo G, Zorec TM, Cerri A, Montani ME (2022). Viral metagenomic data analyses of five new world bat species from Argentina: identification of 35 Novel DNA Viruses. Microorganisms.

[26] de Souza WM, Fumagalli MJ, de Araujo J, Sabino-Santos G, Maia FGM (2018). Discovery of novel anelloviruses in small mammals expands the host range and diversity of the Anelloviridae. Virology.

[27] Myers N, Mittermeier RA, Mittermeier CG, da Fonseca GA, Kent J (2000). Biodiversity hotspots for conservation priorities. Nature.

[28] Brouat C, Tollenaere C, Estoup A, Loiseau A, Sommer S (2014). Invasion genetics of a human commensal rodent: the black rat *Rattus rattus* in Madagascar. Mol Ecol.

[29] Scobie K, Rahelinirina S, Soarimalala V, Andriamiarimanana FM, Rahaingosoamamitiana C (2024). Reproductive ecology of the black rat (*Rattus rattus*) in Madagascar: the influence of density-dependent and -independent effects. Integr Zool.

[30] Amatya R, Deem SL, Porton IJ, Wang D, Lim ES (2017). Complete Genome Sequence of Torque teno indri virus *1*, a novel anellovirus in blood from a free-living lemur. Genome Announc.

[31] Yoder AD, Yang Z (2004). Divergence dates for malagasy lemurs estimated from multiple gene loci: geological and evolutionary context. Mol Ecol.

[32] Poux C, Madsen O, Marquard E, Vieites DR, de Jong WW (2005). Asynchronous colonization of Madagascar by the four endemic clades of primates, tenrecs, carnivores, and rodents as inferred from nuclear genes. Syst Biol.

[33] Steppan SJ, Schenk JJ (2017). Muroid rodent phylogenetics: 900-species tree reveals increasing diversification rates. PLoS One.

[34] Tollenaere C, Brouat C, Duplantier J, Rahalison L, Rahelinirina S (2010). Phylogeography of the introduced species *Rattus rattus* in the western Indian Ocean, with special emphasis on the colonization history of Madagascar. J Biogeogr.

[35] Junge RE, Baden AL (2021). Health assessment of black-and-white ruffed lemurs (*Varecia Variegata*) in ranomafana national park, Madagascar. *J Zoo Wildl Med*.

[36] Junge RE, Louis EE (2005). Preliminary biomedical evaluation of wild ruffed lemurs (Varecia variegata and V. rubra). Am J Primatol.

[37] Paietta EN, Kraberger S, Custer JM, Vargas KL, Espy C (2023). Characterization of diverse anelloviruses, cressdnaviruses, and bacteriophages in the human oral DNA Virome from North Carolina (USA). Viruses.

[38] Paietta EN, Kraberger S, Regney M, Custer JM, Ehmke E (2024). Interspecies papillomavirus type infection and a novel papillomavirus type in red ruffed lemurs (Varecia rubra). Viruses.

[39] Paietta EN, Kraberger S, Custer JM, Vargas KL, Ehmke E (2024). Metagenome-assembled microvirus and cressdnavirus genomes from fecal samples of house mice (*Mus musculus*). *Microbiol Resour Announc*.

[40] Bolger AM, Lohse M, Usadel B (2014). Trimmomatic: a flexible trimmer for Illumina sequence data. Bioinformatics.

[41] Li D, Liu CM, Luo R, Sadakane K, Lam TW (2015). MEGAHIT: an ultra-fast single-node solution for large and complex metagenomics assembly via succinct de Bruijn graph. Bioinformatics.

[42] Buchfink B, Xie C, Huson DH (2015). Fast and sensitive protein alignment using DIAMOND. Nat Methods.

[43] Tisza MJ, Varsani A, Petrosino JF, Cregeen SJJ (2025). Cenote-Taker 3 for Fast and Accurate Virus Discovery and Annotation of the Virome. bioXriv.

[44] Black EJ, Powell CS, Dempsey DM, Hendrickson RC, Mims LR (2026). Virus taxonomy: the database of the International Committee on Taxonomy of Viruses. Nucleic Acids Res.

[45] Katoh K, Standley DM (2013). MAFFT multiple sequence alignment software version 7: improvements in performance and usability. Mol Biol Evol.

[46] Capella-Gutiérrez S, Silla-Martínez JM, Gabaldón T (2009). trimAl: a tool for automated alignment trimming in large-scale phylogenetic analyses. Bioinformatics.

[47] Minh BQ, Schmidt HA, Chernomor O, Schrempf D, Woodhams MD (2020). IQ-TREE 2: new models and efficient methods for phylogenetic inference in the genomic era. Mol Biol Evol.

[48] Stöver BC, Müller KF (2010). TreeGraph 2: combining and visualizing evidence from different phylogenetic analyses. BMC Bioinformatics.

[49] Muhire BM, Varsani A, Martin DP (2014). SDT: a virus classification tool based on pairwise sequence alignment and identity calculation. PLoS One.

[50] Roux S, Adriaenssens EM, Dutilh BE, Koonin EV, Kropinski AM (2019). Minimum information about an uncultivated virus genome (MIUViG). Nat Biotechnol.

[51] Fu L, Niu B, Zhu Z, Wu S, Li W (2012). CD-HIT: accelerated for clustering the next-generation sequencing data. Bioinformatics.

[52] Aroney STN, Newell RJP, Nissen JN, Camargo AP, Tyson GW (2025). CoverM: read alignment statistics for metagenomics. Bioinformatics.

[53] Martin DP, Varsani A, Roumagnac P, Botha G, Maslamoney S (2021). RDP5: a computer program for analyzing recombination in, and removing signals of recombination from, nucleotide sequence datasets. Virus Evol.

[54] Martin D, Rybicki E (2000). RDP: detection of recombination amongst aligned sequences. Bioinformatics.

[55] Padidam M, Sawyer S, Fauquet CM (1999). Possible emergence of new geminiviruses by frequent recombination. Virology.

[56] Martin DP, Posada D, Crandall KA, Williamson C (2005). A modified bootscan algorithm for automated identification of recombinant sequences and recombination breakpoints. AIDS Res Hum Retroviruses.

[57] Smith JM (1992). Analyzing the mosaic structure of genes. J Mol Evol.

[58] Posada D, Crandall KA (2001). Evaluation of methods for detecting recombination from DNA sequences: computer simulations. Proc Natl Acad Sci USA.

[59] Gibbs MJ, Armstrong JS, Gibbs AJ (2000). Sister-scanning: a monte carlo procedure for assessing signals in recombinant sequences. Bioinformatics.

[60] Lam HM, Ratmann O, Boni MF (2018). Improved algorithmic complexity for the 3SEQ recombination detection algorithm. Mol Biol Evol.

[61] Oksanen J, Blanchet FG, Friendly M, Kindt R, Legendre P (2020).

[62] Butts CT (2008). Network: a package for managing relational data in r. J Stat Softw.

[63] Butts CT, Hunter D, Handcock M, Bender-deMoll S, Horner J (2015). Package ‘network’. R package version.

[64] Hunter DR, Handcock MS, Butts CT, Goodreau SM, Morris M (2008). ergm: a package to fit, simulate and diagnose exponential-family models for networks. J Stat Soft.

[65] Krivitsky PN (2012). Exponential-family random graph models for valued networks. Electron J Stat.

[66] Duxbury S (2024). https://cran.r-project.org/web/packages/ergMargins/index.html.

[67] Jarkasi NS, Sekawi Z, Kqueen CY, Othman Z (2018). A review on the global widespread of TTV infection among humans population. Pertanika J Sch Res Rev.

[68] van Elst T, Sgarlata GM, Schüßler D, Tiley GP, Poelstra JW (2025). Integrative taxonomy clarifies the evolution of a cryptic primate clade. Nat Ecol Evol.

[69] Xiong Y-Q, Mo Y, Chen M-J, Cai W, He W-Q (2018). Detection and phylogenetic analysis of torque teno virus (TTV) carried by murine rodents and house shrews in China. Virology.

[70] Merritt JF (2010). The Biology of Small Mammals.

[71] Rahelinirina S, Rajerison M, Telfer S, Savin C, Carniel E (2017). The Asian house shrew Suncus murinus as a reservoir and source of human outbreaks of plague in Madagascar. PLoS Negl Trop Dis.

[72] Martin DP, Biagini P, Lefeuvre P, Golden M, Roumagnac P (2011). Recombination in eukaryotic single stranded DNA viruses. Viruses.

[73] Lefeuvre P, Lett JM, Varsani A, Martin DP (2009). Widely conserved recombination patterns among single-stranded DNA viruses. J Virol.

[74] Haloschan M, Bettesch R, Görzer I, Weseslindtner L, Kundi M (2014). TTV DNA plasma load and its association with age, gender, and HCMV IgG serostatus in healthy adults. Age (Dordr).

[75] Lim ES, Zhou Y, Zhao G, Bauer IK, Droit L (2015). Early life dynamics of the human gut virome and bacterial microbiome in infants. Nat Med.

[76] Kaczorowska J, Deijs M, Klein M, Bakker M, Jebbink MF (2022). Diversity and long-term dynamics of human blood anelloviruses. J Virol.

[77] Kaczorowska J, Cicilionytė A, Timmerman AL, Deijs M, Jebbink MF (2022). Early-life colonization by anelloviruses in infants. Viruses.

[78] Feng AYT, Himsworth CG (2014). The secret life of the city rat: a review of the ecology of urban Norway and black rats (Rattus norvegicus and Rattus rattus). Urban Ecosyst.

[79] Low BW, Mills H, Algar D, Hamilton N (2012). Home ranges of introduced rats on christmas Island: a pilot study. Ecol Manag Restor.

