# Supplementary material for: Host–anellovirus interactions in an island ecosystem: non-human primates and rodents in Madagascar harbour diverse, rich anellovirus populations
Source: Microb Genom. 2026 Apr 9;12(4):001681. doi: 10.1099/mgen.0.001681 (PMC13065334; doi:10.1099/mgen.0.001681)
Supplement: Fig. S1. [file mgen-12-01681-s002.pdf]

Genus	Genus	Species
New genus2	New genus2 new sp2	madalem torque teno virus 2
New genus2	New genus2 new sp3	madalem torque teno virus 1
New genus2	New genus2 new sp1	madalem torque teno virus 3
New genus3	New genus3 new sp1	madanes torque teno virus 1
Aleptorquevirus	*Aleptorquevirus culic1*	madamur torque teno virus 101
Wawtorquevirus	*Wawtorquevirus murid3*	madamur torque teno virus 55
Aleptorquevirus	Aleptorquevirus new sp2	madamur torque teno virus 78
Rhotorquevirus	Rhotorquevirus new sp1	madamur torque teno virus 76
Aleptorquevirus	Aleptorquevirus new sp1	madamur torque teno virus 129
New genus1	New genus1 new sp1	madasor torque teno virus 2

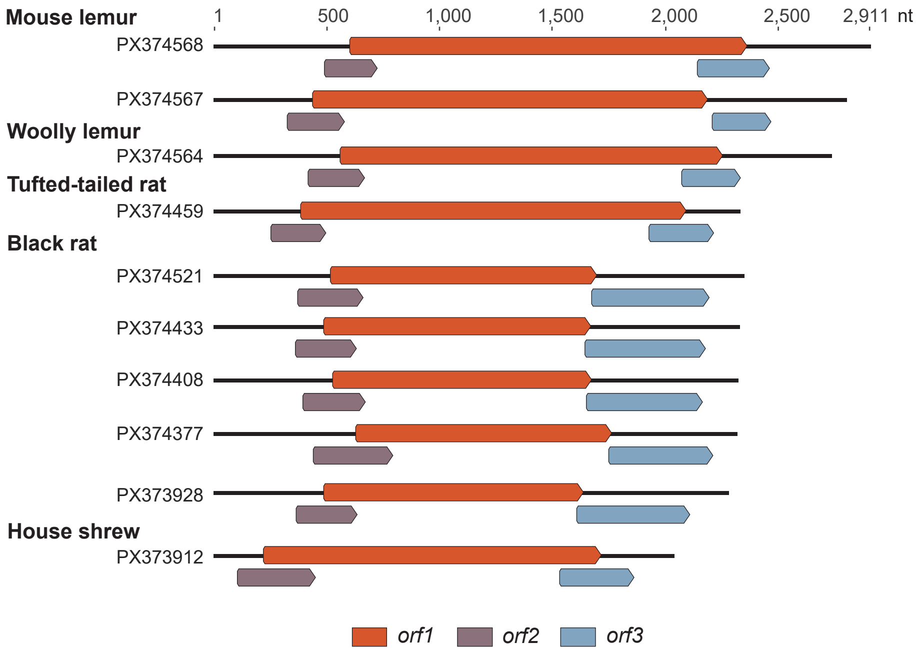

Supplementary Figure 1: Representative genomes of members from each species level grouping of the anelloviruses from this study.
